# Interferon-Alpha Administration Enhances CD8+ T Cell Activation in HIV Infection

**DOI:** 10.1371/journal.pone.0030306

**Published:** 2012-01-24

**Authors:** Maura Manion, Benigno Rodriguez, Kathleen Medvik, Gareth Hardy, Clifford V. Harding, Robert T. Schooley, Richard Pollard, David Asmuth, Robert Murphy, Edward Barker, Kirsten E. Brady, Alan Landay, Nick Funderburg, Scott F. Sieg, Michael M. Lederman

**Affiliations:** 1 Division of Infectious Diseases, Center for AIDS Research, Case Western Reserve University/University Hospitals Case Medical Center, Cleveland, Ohio, United States of America; 2 Department of Pathology, Case Western Reserve University/University Hospitals Case Medical Center, Cleveland, Ohio, United States of America; 3 Division of Infectious Diseases, University of California San Diego, La Jolla, California, United States of America; 4 Division of Infectious Diseases, University of California Davis, Sacramento, California, United States of America; 5 Division of Infectious Diseases, Center for AIDS Research, Northwestern University Feinberg School of Medicine, Chicago, Illinois, United States of America; 6 Department of Immunology and Microbiology, Rush University Medical Center, Chicago, Illinois, United States of America; New York University, United States of America

## Abstract

**Background:**

Type I interferons play important roles in innate immune defense. In HIV infection, type I interferons may delay disease progression by inhibiting viral replication while at the same time accelerating disease progression by contributing to chronic immune activation.

**Methods:**

To investigate the effects of type I interferons in HIV-infection, we obtained cryopreserved peripheral blood mononuclear cell samples from 10 subjects who participated in AIDS Clinical Trials Group Study 5192, a trial investigating the activity of systemic administration of IFNα for twelve weeks to patients with untreated HIV infection. Using flow cytometry, we examined changes in cell cycle status and expression of activation antigens by circulating T cells and their maturation subsets before, during and after IFNα treatment.

**Results:**

The proportion of CD38+HLA-DR+CD8+ T cells increased from a mean of 11.7% at baseline to 24.1% after twelve weeks of interferon treatment (p = 0.006). These frequencies dropped to an average of 20.1% six weeks after the end of treatment. In contrast to CD8+ T cells, the frequencies of activated CD4+ T cells did not change with administration of type I interferon (mean percentage of CD38+DR+ cells = 2.62% at baseline and 2.17% after 12 weeks of interferon therapy). As plasma HIV levels fell with interferon therapy, this was correlated with a “paradoxical” increase in CD8+ T cell activation (p<0.001).

**Conclusion:**

Administration of type I interferon increased expression of the activation markers CD38 and HLA DR on CD8+ T cells but not on CD4+ T cells of HIV+ persons. These observations suggest that type I interferons may contribute to the high levels of CD8+ T cell activation that occur during HIV infection.

## Introduction

HIV-1 infection is characterized by progressive CD4+ T lymphocytopenia. The mechanisms driving this progressive loss of CD4+ T-cells are not completely understood. Several studies have found that increased T cell activation [1]–[3] and turnover [4]–[9] predicts risk of disease progression in HIV infection [10]–[13].

Potential drivers of cellular activation and turnover in chronic HIV infection include the type I interferons. Type I interferons play an important role in innate and adaptive immune defenses against viral replication and in immune cell maturation [14], [15]. In HIV infection, although interferon levels in blood have been difficult to detect and may vary with the stage of infection [16], interferon exposure is implicated, as microarray studies have shown substantial upregulation of interferon stimulated gene expression [17]–[21]. Type 1 interferons can induce cellular resistance to HIV propagation as well as enhanced cytolytic defenses [22]–[28].

The antiviral activities of type I interferons have been exploited for the systemic treatment of hepatitis B and C infection [29]. AIDS Clinical Trial Group (ACTG) Study 5192 was designed to evaluate the safety and antiretroviral activity of type I interferon administration in chronic HIV infection. We decided to use this opportunity (through ACTG New Works Concept Sheet 289) to investigate the effects of exogenous type I interferon administration on indices of immune activation and CD4 T cell turnover, as well as its effects on innate immunity, particularly natural killer cell frequency in HIV-infected persons. We found that systemic administration of interferon-alpha induces phenotypic markers of immune activation (CD38 and HLA-DR) on CD8+ T cells, but not on CD4+ T cells. We also saw a tendency for CD4+ central memory T cells to enter cell cycle during interferon therapy, although this trend did not reach statistical significance.

## Methods

### Study Population

These studies were approved by the institutional review boards at University of California, Davis, Northwestern University and Duke University. De-identified stored specimens were examined at Case Western Reserve University. Before study initiation, all study participants provided written informed consent by. All clinical investigation was conducted according to the principles expressed in the Declaration of Helsinki.

In order to investigate the effects of interferon in HIV-infection, we obtained cryopreserved peripheral blood mononuclear cell (PBMC) samples from AIDS Clinical Trial Group (ACTG) study 5192 (n = 13, Table 1), a trial investigating the activity of systemic administration of type I interferon to persons with untreated HIV infection. In this open label study, patients received 12 weekly intramuscular injections of 180 ug of pegylated IFN-alpha 2a (Genentech (Roche), San Francisco, CA) and were then followed for 6 weeks after interferon treatment ended. Patients eligible to enroll in ACTG 5192 had a CD4+ T cell count of >300 cells/ul, had a plasma HIV-1 RNA level >5000 copies/ml, were antiretroviral therapy-naïve or had not received antiretroviral therapy for at least twelve weeks, had no evidence of infection with hepatitis B or C virus and had serum transaminase levels no greater than grade 1 by ACTG toxicity tables at entry. Exclusion criteria included a history of severe psychiatric illness or a history of chronic illness that could be worsened by interferon therapy [21].

**Table 1:** Patient Cohort from AIDS Clinical Trial Group Study 5192.

Patient ID	Sex	Age	CD4+ T Cell Count (cells/ul)	PlasmaHIV RNA (log10 copies/mL)
1	M	35	367.5	4.37
2	M	45	480	4.49
3	M	37	929.5	4.10
4	M	46	650	3.57
5	M	37	372	4.39
6	M	42	325.5	4.72
7	M	30	298	3.86
8	M	32	612	3.84
9	M	45	359.5	4.76
10	F	36	510	4.56

Peripheral blood mononuclear cells (PBMC) were prepared and viably cryopreserved before the interferon treatment was initiated (week 0), during the course of treatment (weeks 3 and 12), and 6 weeks after treatment was completed (week 18). For studies involving natural killer (NK) cells, samples were obtained at Rush Medical Center from five healthy donors not known to be HIV infected.

### Immunologic Analyses

Cryopreserved PBMC were thawed and stained with fluorochrome-labeled monoclonal antibodies for flow cytometric analyses. Allophycocyanin (APC) anti-CD4, Peridinin-cholorphyll protein (PerCP) anti-CD8, Fluorescein isothiocyanate (FITC) anti-HLA-DR, Phycoerythrin (PE) anti-CD38, FITC anti-CD45RA, PE-Cyanin 7 (PE-Cy7) anti-CCR7, Pacific Blue anti-CD4, APC anti-CD27 were purchased from Becton Dickinson Biosciences (San Jose, CA). Fluorochrome-matched isotype control antibodies were used to establish background staining and gating.

For examination of intracellular Ki-67 expression, samples were fixed and permeabilized using a commercial kit (Cytofix CytoPerm, Becton Dickinson Biosciences) and then stained with PE-anti-Ki67 (Becton Dickinson, San Jose, CA) at 4°C for 30 min. Fluorochrome-matched isotypes were used to establish background staining and proper gating of cells expressing this marker.

We examined indices of immune activation by studying cell cycling (Ki-67 expression), expression of activation antigens (CD38 and HLA-DR), and survival of phenotypically-defined central memory CD4+ T cells. Central memory cells were defined as CD 27+CD45RA− and CCR7+, naïve cells as CD27+, CD45RA+, and CCR7+, transitional memory cells as CD27+, CD45RA−, and CCR7−, and effector memory cells as CD27−, CD45RA−, and CCR7−. We gated for live lymphocytes based upon cell size and granularity, and we aimed to collect a million events (a minimum of 100,000 events were counted for each sample) using an LSR II flow cytometer (Becton Dickinson). For examination of natural killer cell frequency, cryopreserved PBMCs were thawed and cultured overnight in RPMI 1640 (BioWhittaker, Walkersville, MD) supplemented with 10% heat-inactivated fetal bovine serum (GemCell), 100 U/mL penicillin, 100 ug/mL streptomycin (Sigma, St. Louis, MO) and 2 mM L-glutamine (Sigma). PBMCs were washed, resuspended in phosphate buffered saline (PBS) containing 0.5% bovine serum albumin (Sigma) and 0.1% sodium azide (FACS buffer), and stained for viability using AquaLiveDead (Invitrogen, Carlsbad, CA). Nonspecific antibody binding to Fc receptors was blocked by incubation of the cells with Fc-receptor blocking reagent (Miltenyi Biotec, Auburn, CA). PBMCs were then surface stained with the following antibodies: anti-CD3 Pacific Blue, anti-CD14 Pacific Blue, and anti-CD16 Pe-Cy7 (BD Pharmingen). In addition, anti-CD19 Pacific Blue (Biolegend), anti- CD56 FITC (BD Biosciences), and anti-CD69 APC (BD Biosciences) were used. After incubation, cells were washed with FACS buffer, fixed with 2% formaldehyde, and stored at 4°C until analysis on a BD LSRII flow cytometer. NK cells were gated on viable CD3-CD14-CD20- lymphocytes, and the different NK cell populations CD56dimCD16neg, CD56brightCD16neg, CD56dimCD16pos, CD56brightCD16neg, and CD56negCD16pos were analyzed using FACS Diva version 6.1.1 software.

### Statistical Methods

We used conventional measures of central location and spread to describe the data. We used the Wilcoxon signed-ranks test for bivariate comparisons of readouts at different timepoints. To assess increasing or decreasing trends during the interferon treatment period, we fitted repeated-measures general linear models for each readout during the on-interferon period, and we used mixed effects models to assess the contribution of multiple readouts to changes in immune activation during the study period. Data were analyzed using SPSS Statistics v. 20.0, IBM Corp, Somers, NY and Stata MP v. 11.1, Stata Corp, College Station, TX. All tests of hypothesis are 2-sided without formal correction for multiple comparisons, and a p-value of ≤0.05 was considered significant.

## Results

ACTG 5192 enrolled thirteen HIV-1 infected volunteers. Two participants discontinued therapy during the course of the trial [21]. We were able to study samples obtained from ten of the eleven patients who completed the trial. One sample could not be analyzed due to poor cell viability. Table 1 shows the baseline characteristics of the ten participants we studied.

### Effects of interferon therapy on circulating CD4 and CD8 T cell numbers and plasma HIV RNA levels

**Figure 1:**
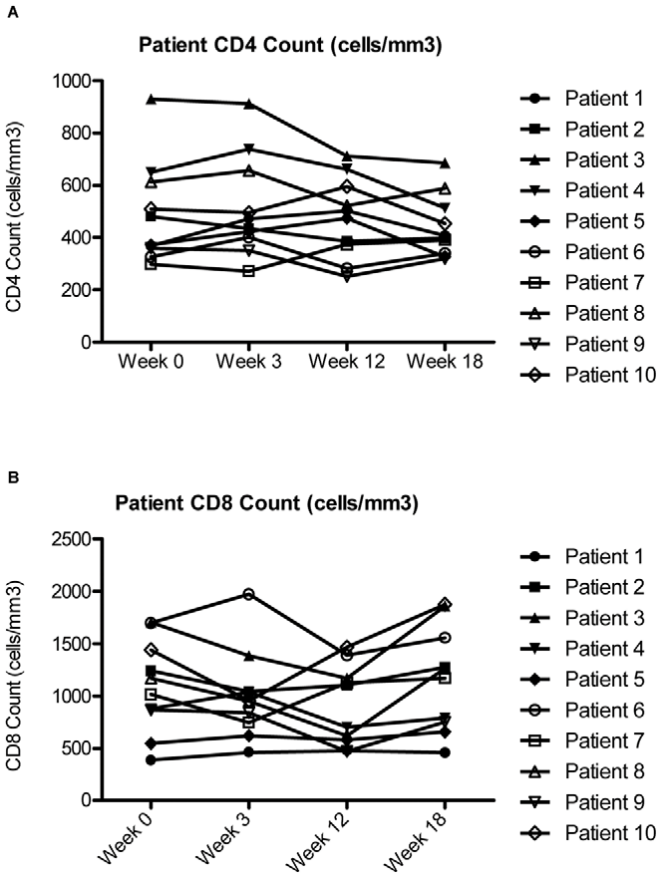
CD4+ and CD8+ T cell counts. The CD4 Cell Count (A) and the CD8 Cell Count (B) are portrayed over the course of interferon-alpha treatment during the ACTG 5192 Study. Week 0 corresponds to the patients' baseline cell counts before beginning Interferon-Alpha Therapy. Weeks 3 and 12 correspond to 3 weeks and 12 weeks of interferon-alpha Therapy. Interferon-Alpha therapy was stopped at Week 12, and therefore Week 18 corresponds to 6 weeks off therapy.

Over the course of interferon therapy, there was no significant change in the numbers of circulating CD4 and CD8 cells at the time points studied (Figure 1). There were, however, significant increases in CD4+ T cell percentages early in treatment during weeks 2 and 4 when the greatest declines in plasma HIV levels were observed [21]. As previously reported [21], plasma HIV RNA levels decreased significantly from week 0 (24,024 copies/mL) to week 3 (3,142 copies/mL) (p = 0.02) and from week 0 to week 12 (8090 copies/mL) (p = 0.039) during interferon therapy, and there was a significant increase (p = 0.02) in HIV RNA levels to 28,772 copies/mL six weeks after therapy was stopped.

### CD4+ T cell Maturation Subsets

At baseline, 45% of CD4 T cells were phenotypically naïve, 20% were central memory cells, 13% were transitional memory cells and 7 percent were effector memory cells. Administration of interferon-α did not alter the proportions of CD4+ T cell maturation subsets (Table 2).

**Table 2:** CD4+ T cell Dynamics.

	Week 0	Week 3	Week 12	Week 18
**Central Memory T cells**	20%	19%	22%	20%
**Naive T cells**	45%	43%	44%	46%
**Transitional Memory T cells**	13%	13%	16%	15%
**Effector Memory T cells**	7%	6%	5%	5%

The proportions of CD4+ T cell maturation subsets are shown over the course of interferon-alpha treatment during the ACTG 5192 Study. Central memory cells were defined as CD27+CD45RA− and CCR7+, whereas naïve cells are CD27+, CD45RA+, and CCR7+, transitional memory cells are CD27+, CD45RA−, and CCR7−, and effector memory cells are CD27−, CD45RA−, and CCR7−. Week 0 corresponds to the patients' baseline cell counts before beginning Interferon-Alpha Therapy. Weeks 3 and 12 correspond to 3 weeks and 12 weeks of interferon-alpha therapy. Interferon-Alpha therapy was stopped at Week 12, and therefore Week 18 corresponds to 6 weeks off therapy.

### Indices of Immune Activation

**Figure 2:**
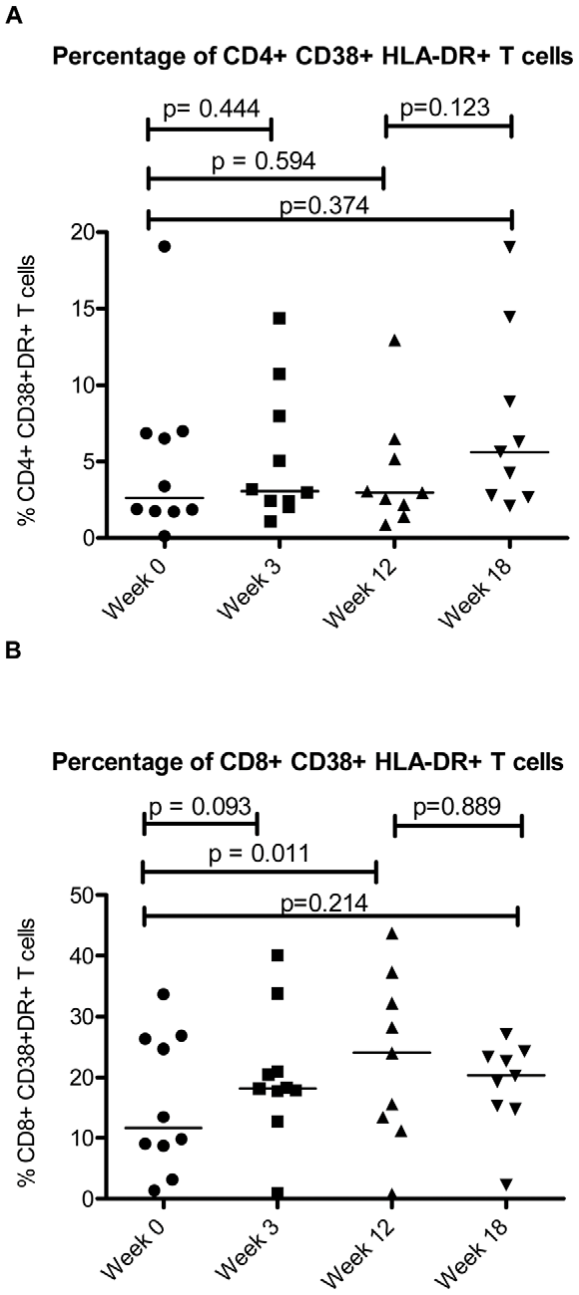
Co-expression of CD38 and HLA-DR on CD4+ and CD8+ T cells. The percentage of CD4+ T cells (A) and CD8+ T cells (B) co-expressing CD38 and HLA-DR is shown over the course of interferon-alpha treatment during the ACTG 5192 Study. Week 0 corresponds to the patients' baseline cell counts before beginning Interferon-Alpha Therapy. Weeks 3 and 12 correspond to 3 weeks and 12 weeks of interferon-alpha Therapy. Interferon-Alpha therapy was stopped at Week 12, and therefore Week 18 corresponds to 6 weeks off therapy. The bar represents the median value.

Activation and turnover of T cells has been implicated as central to the pathogenesis of immune deficiency in HIV infection [4], [9], [30]–[32]. Since expression of activation markers CD38 and HLA-DR on circulating T cells is a validated predictor of disease progression in chronic HIV-1 infection [10], we asked if interferon exposure would increase expression of one or both of these markers *in vivo* as it does *in vitro* [33]. The proportion of CD8+ T cells that co-expressed CD38 and HLA-DR tended to increase during interferon administration from a median of 11.7% at baseline to 18.2% after three weeks of interferon treatment (p = 0.093) and increased significantly to 24.1% after twelve weeks of interferon treatment (p = 0.011). The proportion of CD8+ T cells co-expressing CD38 and HLA-DR dropped to 20.1% six weeks after treatment was terminated, and this was not significantly different from baseline (p = 0.214) (Figure 2).

The proportions of CD8+ T cells that were HLA-DR+ but CD38− decreased from a median of 34.8% to 28.7% after three weeks of interferon treatment (p = 0.009) and to 23.0% after twelve weeks of treatment (p = 0.008). These frequencies returned to a median of 38.9% six weeks after treatment ended, which was not a significant change from baseline (p = 0.139). The increase in the proportion of activated (CD38+ HLA-DR+) CD8 cells appeared to be a result of increasing expression of CD38 on CD8 T cells that were already expressing HLA-DR. The proportions of CD8+ T cells that were HLA-DR+ had a non-significant decrease from a median of 55.3% to 50.3% after three weeks of treatment (p = 0.1602) and stayed relatively stable at 51.2% after twelve weeks of treatment (p = 0.2031). The median proportion of HLA-DR+ cells increased to a median of 62.3% six weeks after treatment ended (p = 0.055).

Interferon therapy did not increase significantly the proportions of CD8+ T cells expressing CD38 in the absence of HLA-DR, as the median proportion of these cells was 3.7% at baseline and 5.0% after three weeks of interferon treatment (p = 0.169) and 6.1% after twelve weeks of treatment (p = 0.214). These frequencies tended to fall to a median of 1.2% six weeks after treatment ended (p = 0.086 when compared to baseline).

In contrast to our findings for CD8+ T cells, the frequencies of activated CD4+ T cells did not change with administration of type I interferon (median percentage of CD38+DR+ cells = 2.6% at baseline and 2.2% after 12 weeks of interferon therapy). A non-significant increase in this frequency to a median of 5.6% (p = 0.374 when compared to baseline) was seen 6 weeks after interferon treatment was stopped (Figure 2). Therapy was also not associated with an increase in single positive (CD38+) or (HLA-DR+) CD4 T cells.

To further delineate the effect of interferon on activation indices given the effects of interferon on both viremia and CD8+ T cell activation, we fitted mixed effects models to assess the contribution of viremia reduction on immune activation indices. We found that the magnitude of change in activation during interferon treatment was significantly associated with the change in viremia during the same period (p<0.001).

### Cell Cycling

Since the activation and turnover of CD4+ T cells and especially central memory CD4+ T cells is thought to play a critical role in the progressive immune deficiency of HIV infection, we identified the proportion of cells in CD4+ T cell maturation subsets that were in cell cycle by staining for intra-nuclear expression of Ki-67 at baseline, 3 weeks of therapy, 12 weeks of therapy, and 6 weeks after interferon treatment was ended.

**Figure 3:**
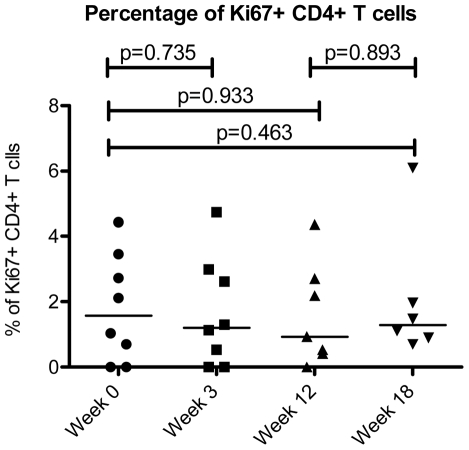
Cell cycle status of CD4+ T cells. The cell cycle status of CD4+ T cells as measured by Ki-67 expression is shown over the course of interferon-alpha treatment during the ACTG 5192 Study. Week 0 corresponds to the patients' baseline cell counts before beginning Interferon-Alpha Therapy. Weeks 3 and 12 correspond to 3 weeks and 12 weeks of interferon-alpha Therapy. Interferon-Alpha therapy was stopped at Week 12, and therefore Week 18 corresponds to 6 weeks off therapy. The bar represents the median value.

When the expression of Ki-67 was evaluated among all circulating CD4+ T cells, there was no indication that this population as a whole was driven into cell cycle by interferon therapy. The proportion of Ki67+ CD4+ T cells remained stable throughout the study period (median values of 1.6% at baseline, 1.2% after three weeks of therapy, 0.9% after twelve weeks of therapy, and 0.9% six weeks after the end of therapy) (Figure 3).

**Figure 4:**
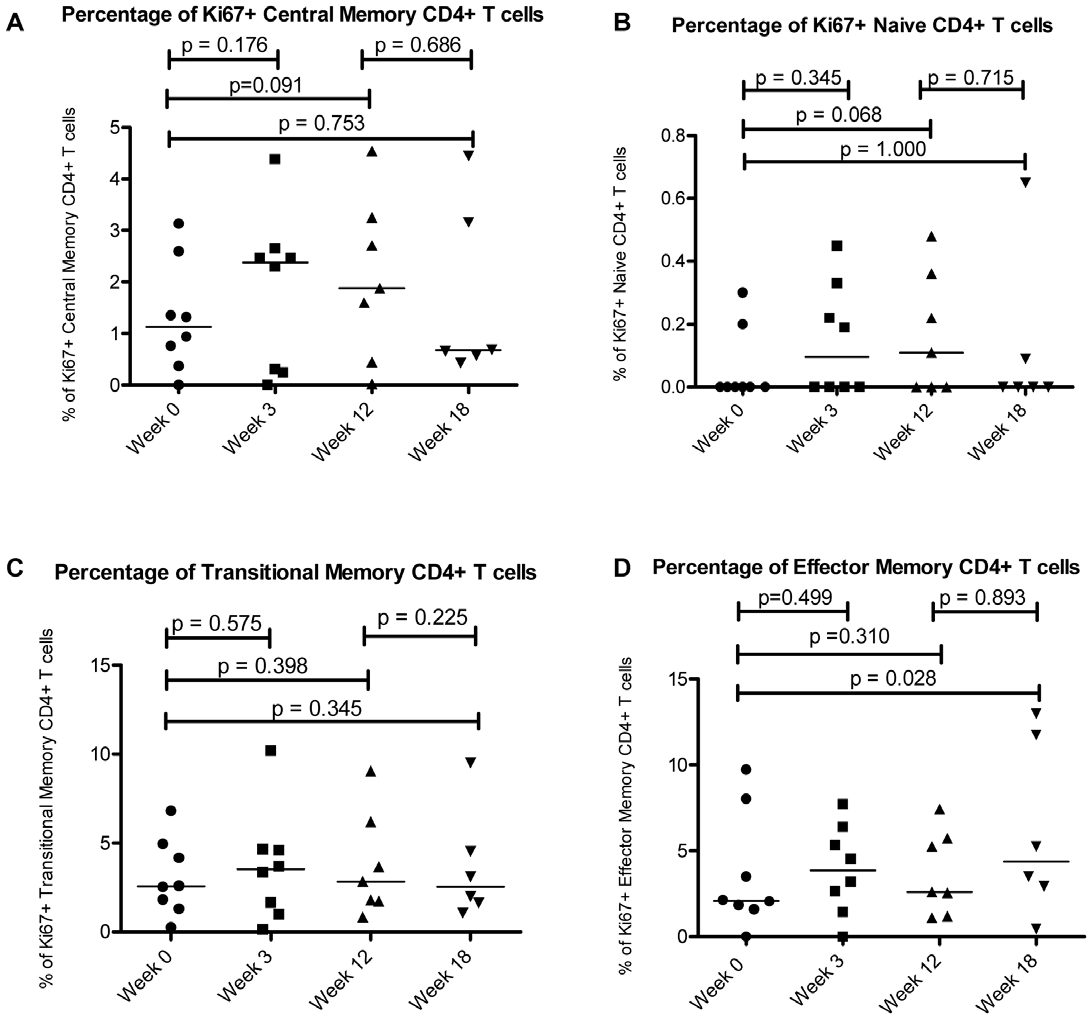
Cell cycle status of CD4+ T cell maturation subsets. The cell cycle status of CD4+ T cell maturation subsets, Central Memory CD4 +T Cells (A), Naive CD4+ T cells (B), Transitional Memory CD4+ T cells (C), and Effector Memory CD4+ Tcells (D), is shown over the course of interferon-alpha treatment during the ACTG 5192 Study. Central memory cells were defined as CD 27+CD45RA− and CCR7+, whereas naïve cells are CD27+, CD45RA+, and CCR7+, transitional memory cells are CD27+, CD45RA−, and CCR7−, and effector memory cells are CD27−, CD45RA−, and CCR7−. Week 0 corresponds to the patients' baseline cell counts before beginning Interferon-Alpha Therapy. Weeks 3 and 12 correspond to 3 weeks and 12 weeks of interferon-alpha therapy. Interferon-Alpha therapy was stopped at Week 12, and therefore Week 18 corresponds to 6 weeks off therapy. The bar represents the median value.

Nonetheless, when expression of Ki-67 was evaluated among defined CD4+ T cell maturation subsets, there was indication that exogenous interferon differentially regulated cycling of cells in these subsets. Interferon therapy tended to increase the proportion of Ki-67+ cells in central memory CD4+ T cells from 1.1% at baseline to 2.4% at week 3 (p = 0.176) and 1.9% at week 12 (p = 0.091, (Figure 4), although these trends failed to reach statistical significance. The proportion of Ki67+ CD4+ naïve cells at baseline was very low and remained low throughout therapy. The proportions of Ki67+ CD4+ effector memory and transitional memory T cells remained stable throughout the twelve weeks of interferon therapy.

### Natural Killer Cells

Natural killer cells respond to interferon-alpha with increased cytolytic capability [27], [28] even in HIV- infected patients [34], [35]. Here we asked if interferon-alpha treatment had any affect on circulating NK cell frequencies. The percentage of CD56bright CD16dim NK cells in HIV-infected patients at baseline was not significantly different from what was seen among healthy donors. Interferon-alpha treatment led to a slight increase in the percentage of CD56brightCD16neg NK cells, although these changes were not statistically significant. The proportion of CD56dimCD16pos NK cells in the infected individuals at baseline was significantly (p = 0.004) lower (30%) than among seronegative donors. Interferon treatment did not affect these proportions during the course of treatment. In addition, CD56negCD16pos NK cells were present in the infected subjects even though they were absent in the seronegative donors, as expected [36]. Interferon-alpha treatment did not affect the percentage of CD56negCD16pos NK cells. In summary, we could find no effect of interferon alpha administration on the proportions of NK cells or their subsets in HIV infection (not shown).

## Discussion

The role and effects of type I interferons in chronic HIV infection are not fully understood. On the one hand, IFN-α effectively inhibits viral replication [22]–[26], [37]–[40]; on the other hand, high level expression of IFN-α could plausibly drive disease progression by activating, exhausting and depleting CD4+ T cells [34]–[37], [41]–[44]. While in some *in vitro* models IFN-α can drive CD4+ T cell apoptosis and death by upregulating TNF related apoptosis inducing ligand (TRAIL) expression in CD4+ cells [41]–[44], in others IFN-α may protect T cells from apoptosis [33], [45]–[48]. We have recently reported that interferon-α may protect T cells from apoptosis *in vitro* [33], conceivably through mechanisms related to upregulation of survival genes [45]–[47]. Thus, the *in vivo* effects of interferon-α may be complex and difficult to predict.

As reported in the primary publication of this study [21], systemic administration of type I interferon resulted in suppression of HIV replication as had been seen in previous studies [22]–[26]. The goal of this substudy was to examine the immunologic effects of type I interferon administration to persons with chronic HIV infection and without HCV co-infection. In earlier studies, decreases in absolute CD4+ T cell counts were seen during interferon administration, but we found no significant change in CD4+ T cell numbers over twelve weeks of interferon therapy. This differs from results of earlier studies, which showed diminished cell counts in subjects with Kaposi's sarcoma (and presumably co-infected with HHV-8) during IFN-α therapy [49]–[51]. One of the major differences between this study and the earlier studies was the use of peglyated interferon alpha-2a Pegylated interferon-α has an increased half-life, providing a longer antiviral effect than the nonpegylated forms [52], [53]. Pegylated interferon alpha- 2a also has a more favorable safety profile than the previously available interferon-alpha formulations [54]. Conceivably, the stability in CD4+ T cell counts we observed may be attributable to differences in disease stage during the clinical trials, as well as the more stable pharmacodynamics of this drug.

High level T cell activation, as reflected in increased surface expression of CD38 and HLA-DR on both CD4+ and CD8+ T cells, is characteristic of untreated HIV infection [1]–[4], [10]–[13]. Treatment with combination antiretroviral therapy characteristically results in prompt and substantial decreases in indices of immune activation [55], [56]. Although this study was small, we found no evidence for a decrease in any of the activation indices we measured. In a study of 11 patients with an average decrease in plasma viremia of 1 log_10_ after antiretroviral therapy, a significant and persistent decrease in immune activation was noted as soon as 2 weeks after treatment initiation that persisted for at least 12 weeks [57]. Yet in this study, with administration interferon-α and an approximate1 log_10_ decrease in plasma HIV RNA levels, we saw no decrease in immune activation indices. To the contrary, we found that systemic administration of type I interferon to persons with untreated HIV infection resulted in increased expression of the activation marker CD38 on CD8+ T cells but not on CD4+ T cells. We did not observe increases in the expression of HLA-DR among either CD4+ or CD8+ T cells. In earlier work, we had found that *in vitro* exposure to interferon-alpha increased surface expression of CD38 on CD8+ T cells but not CD4 T+ cells [33]. We had also found a significant decrease in the proportion of CD38− HLA-DR+ CD8+ T cells during treatment with interferon-α. Several earlier reports have linked the frequency or function of CD38− HLA-DR+ CD8 T cells to a favorable outcome in HIV infection [58]–[60]. These observations suggest that increased endogenous expression of type I interferons may contribute to the high levels of CD38 expression on CD8+ T cells that are characteristic of HIV infection and are recognized predictors of disease progression [10], but that other phenotypic markers of immune activation seen in HIV infection may not be attributable directly to type I interferon exposure.

We found a trend towards increases in proportions of phenotypically defined central memory CD4+ T cells that were in cell cycle during administration of interferon-alpha. Turnover and cycling of central memory CD4+ T cells is thought to be a critical determinant and predictor of outcome in chronic HIV and SIV infection [9], [30]–[32]. With only 10 subjects studied, we may have been underpowered to reveal a significant increase in cell cycling with interferon therapy. A very modest increase in naïve CD4+ T cell cycling also was observed, but the numbers of cycling naïve cells was too low to evaluate quantitative differences in cycling over the course of the study. There is reason to suspect that some effects of exogenous interferon administration in HIV infection might be attenuated as a result of diminished responsiveness to type I interferon, as we had earlier found that monocytes in HIV infection had both decreased type I interferon receptor expression and decreased signaling after interferon exposure [61]. Thus, even a modest effect of exogenous type I interferon on phenotypic indices of immune activation and cell turnover may underestimate the effects of sustained endogenous type I interferon exposure that is suggested by the demonstrable and broad upregulation of interferon responsive genes in chronic HIV infection [17], [20].

Our findings suggest that type I interferon may contribute to the heightened immune activation profile seen in chronic HIV infection but that the activation phenotype in chronic infection is not precisely recapitulated by systemic interferon administration. While some of this may be related to relative refractoriness to additional interferon stimulation, it is also possible that other factors may also contribute to the increased immune activation profile that is characteristic of untreated HIV infection. Some of this may be a consequence of HIV replication itself [62]–[66], or increased antigenic stimulation by HIV peptides or by peptides of other microbes that have been permitted to replicate in the setting of HIV related immune deficiency [62]–[67]. Indeed, our findings suggest that the effect on interferon on CD8+ T cell activation mirrors and may be related to interferon-induced decreases in plasma viremia. We propose that the equilibrium between endogenous interferon levels in response to HIV replication was perturbed by our treatment strategy such that a decrease in HIV levels induced by exogenous interferon resulted in a “paradoxical” increase in immune activation as viremia fell. Recent work by members of our group and others has implicated translocation of microbial products across the damaged gut mucosa as an important driver of immune activation and inflammation in chronic HIV infection [49]–[54], [68]–[73]. While type I interferons are inducible by these microbial TLR agonists [74], broad induction of a variety of inflammatory cytokines [75] is also an expected outcome of systemic exposure to microbial products, and these cytokines may also contribute to immune activation in HIV infection.

In summary, exogenous administration of interferon-alpha to HIV-infected persons can recapitulate and/or exacerbate certain but not all immunophenotypic abnormalities characteristic of untreated HIV infection. It is likely that other factors also contribute to the activation/inflammatory phenotype that characterizes untreated HIV infection.
